# Supplementary figures and images for: Transcriptomic Signature of the Simulated Microgravity Response in Caenorhabditis elegans and Comparison to Spaceflight Experiments
Source: Cells. 2023 Jan 10;12(2):270. doi: 10.3390/cells12020270 (PMC9856674; doi:10.3390/cells12020270)

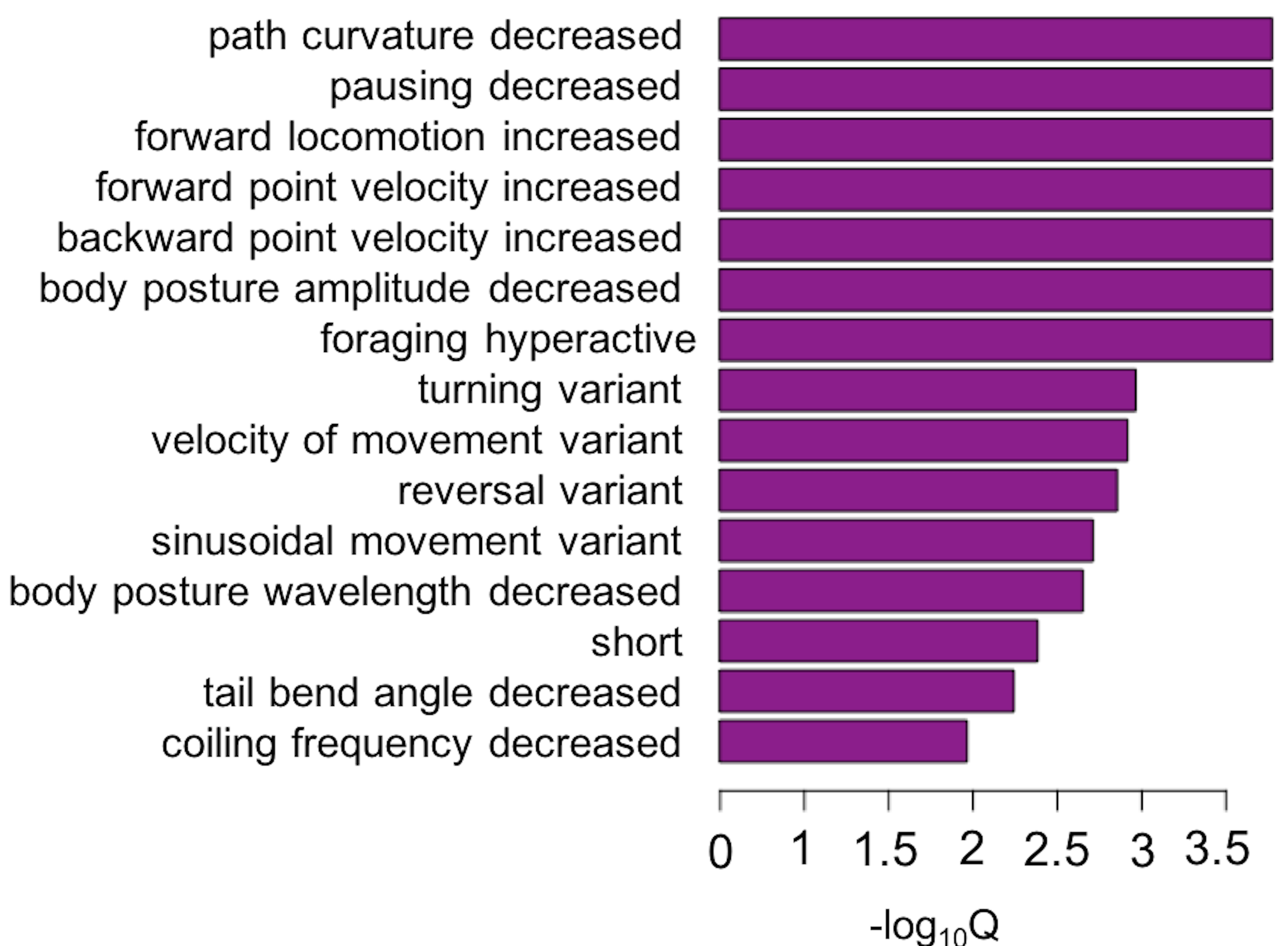

Supplement: Supplementary file 1 [file cells-12-00270-s001.zip › FigS1.png]

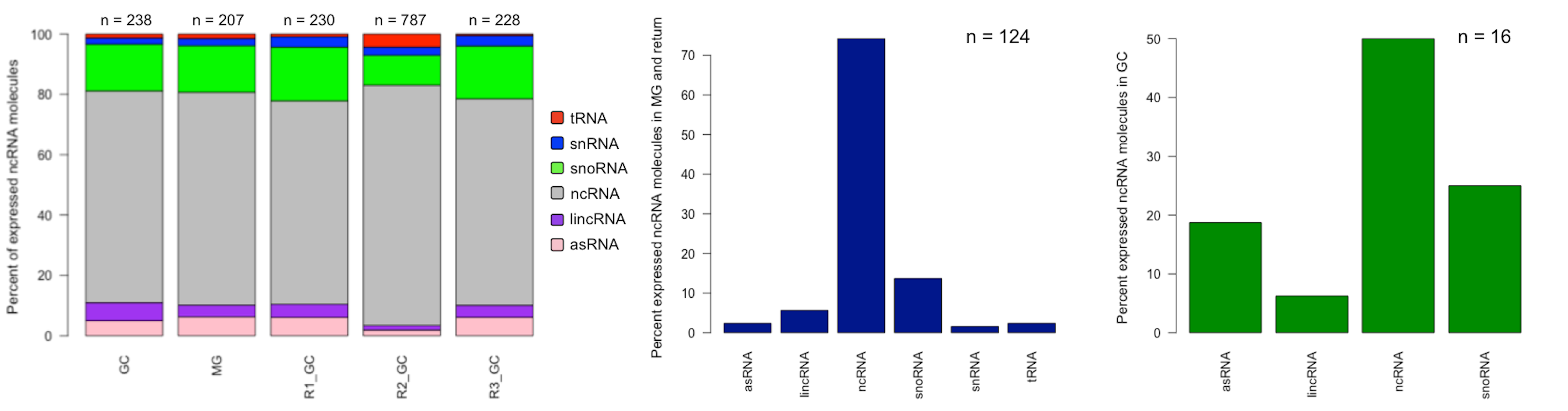

Supplement: Supplementary file 1 [file cells-12-00270-s001.zip › FigS2.png]

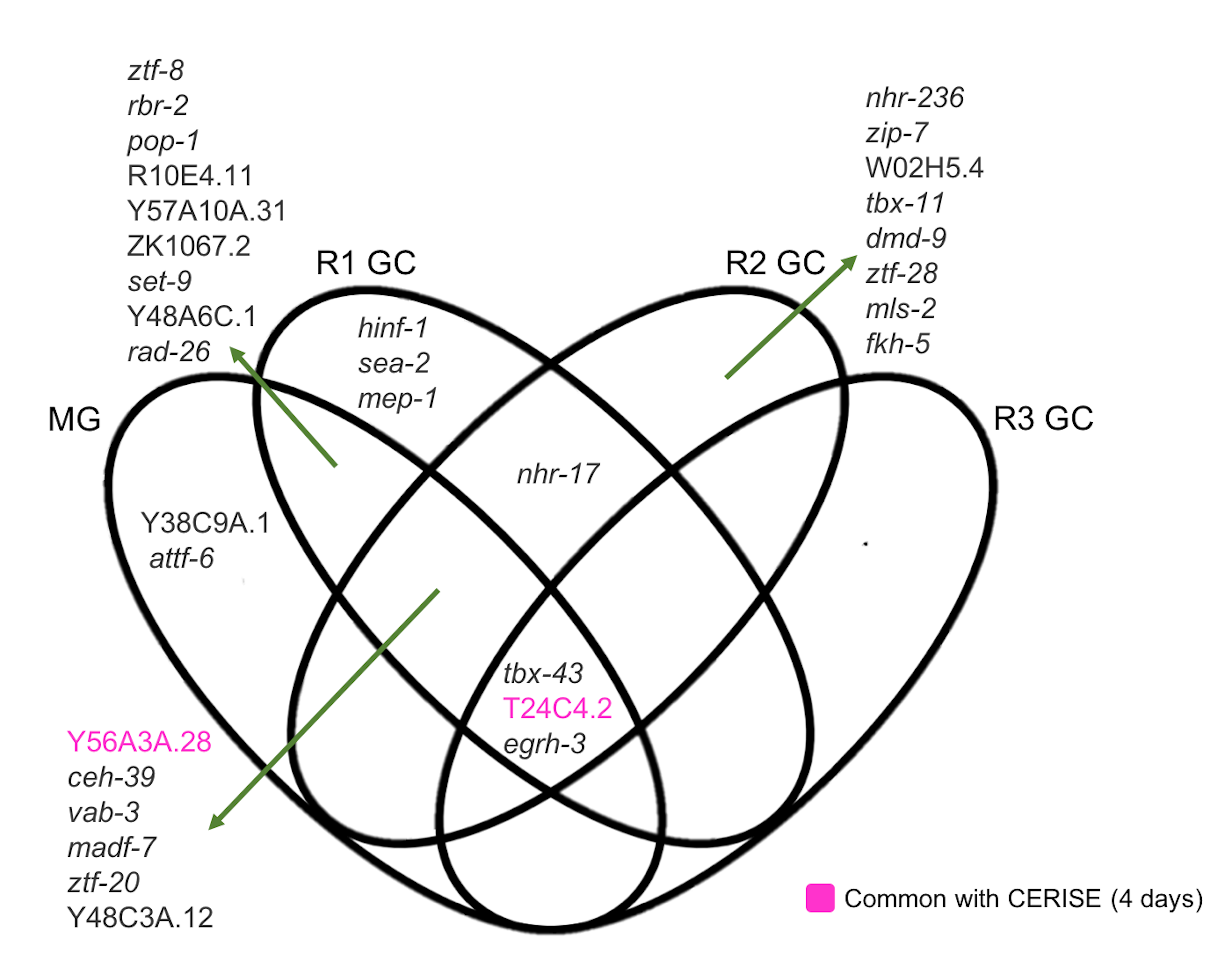

Supplement: Supplementary file 1 [file cells-12-00270-s001.zip › FigS3.png]

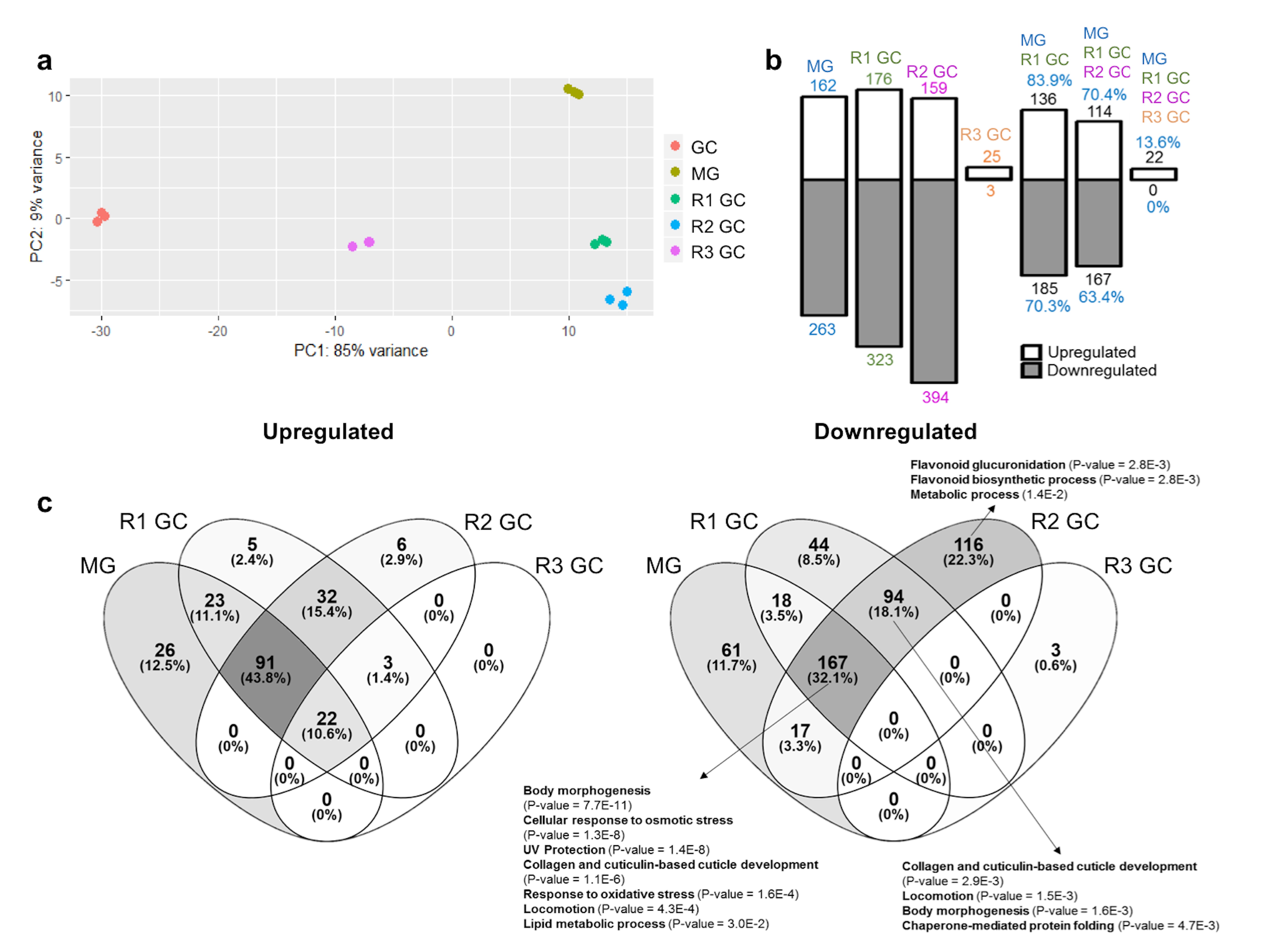

Supplement: Supplementary file 1 [file cells-12-00270-s001.zip › FigS4.png]

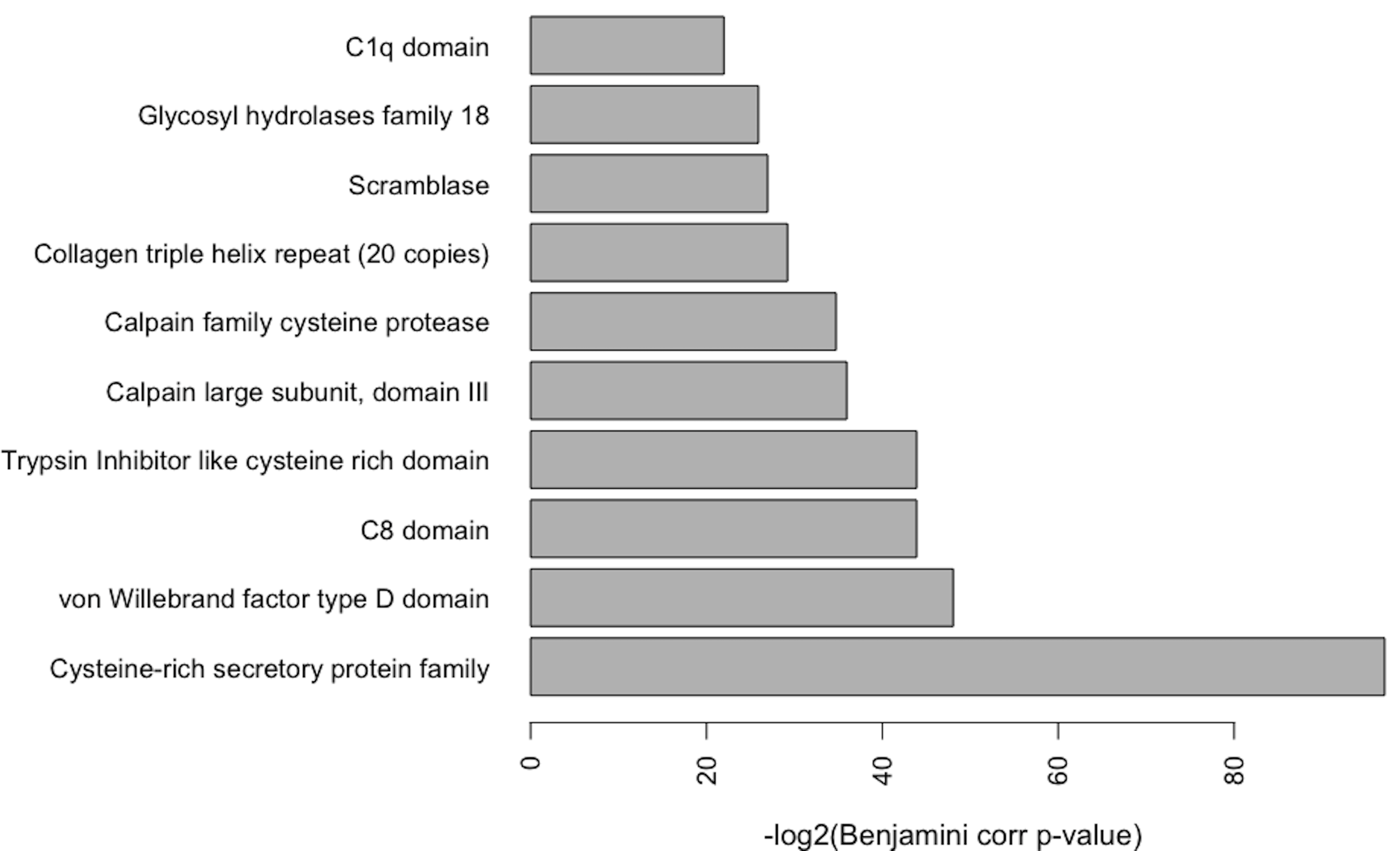

Supplement: Supplementary file 1 [file cells-12-00270-s001.zip › FigS5.png]

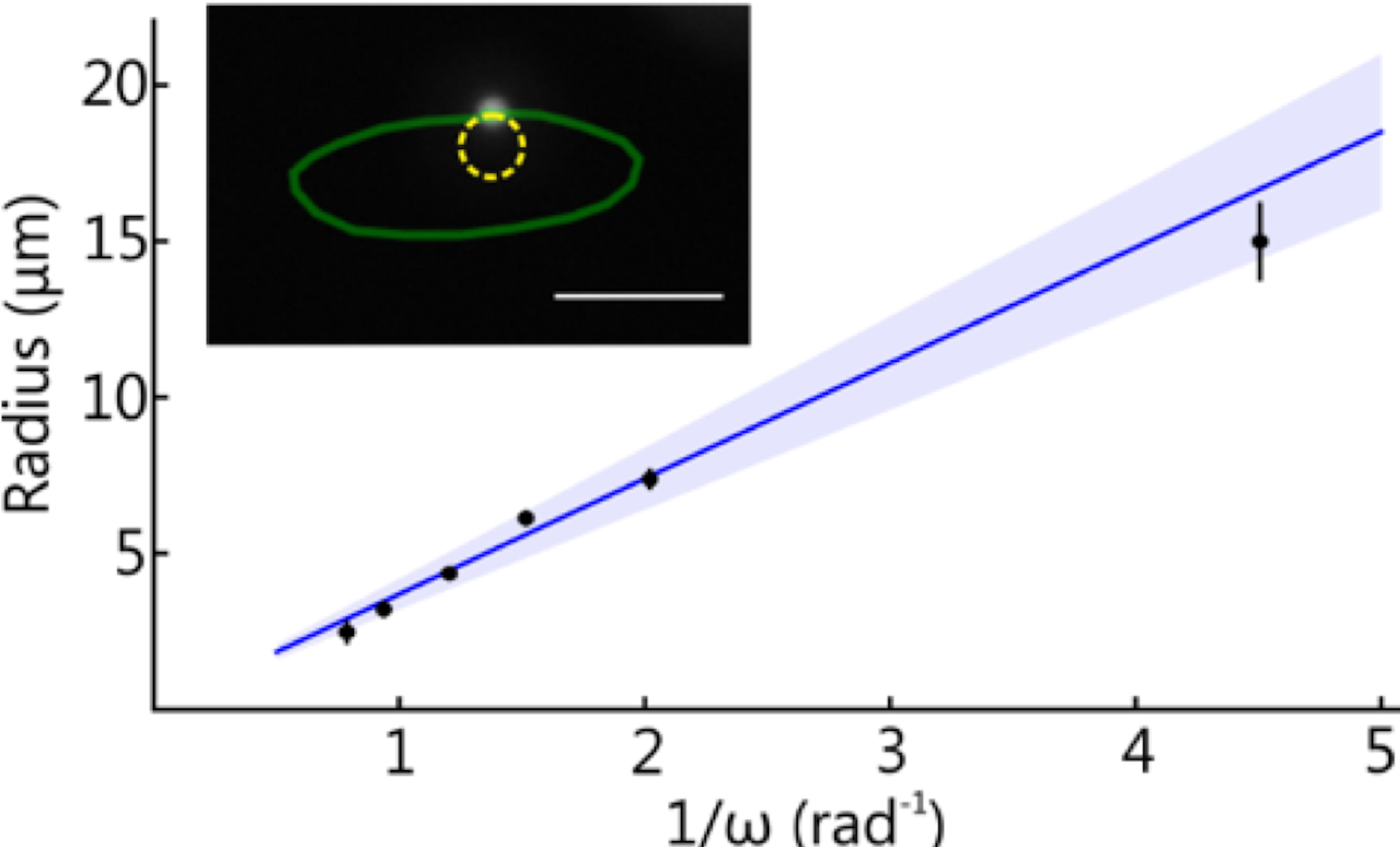

Supplement: Supplementary file 1 [file cells-12-00270-s001.zip › FigS6.png]
